# Anomalies in the valve morphogenesis of the centric diatom alga *Aulacoseira islandica* caused by microtubule inhibitors

**DOI:** 10.1242/bio.035519

**Published:** 2018-07-30

**Authors:** Yekaterina Bedoshvili, Ksenia Gneusheva, Maria Popova, Alexey Morozov, Yelena Likhoshway

**Affiliations:** Limnological Institute of the Siberian Branch of the Russian Academy of Sciences, Irkutsk 664033, Russia

**Keywords:** Morphogenesis, Diatom, Valve, Girdle bands, Colchicine, Paclitaxel, Cytokinesis

## Abstract

Of all unicellular organisms possessing a cell wall, diatoms are the most adept at micro- and nanoscale embellishment of their frustules. Elements of their cell walls are formed inside the cell under cytoskeletal control. In this work, we used laser scanning microscopy and electron microscopy to describe the major stages of cell wall formation in the centric diatom algae *Aulacoseira islandica* and to study the effect of various microtubule inhibitors on the morphogenesis of frustule elements. Our results show that colchicine inhibits karyokinesis and cytokinesis in *A. islandica* colonies. In contrast, valve morphogenesis is changed, rather than inhibited altogether. In normal cells, this process starts simultaneously in both daughter cells, beginning with the formation of two adjacent discs that later become valve faces and connecting spines. Under colchicine treatment, however, the cleavage furrow is blocked and a single lateral valve forms on the side of the cylindrical frustule. As a result, a single hollow pipe forms instead of two separate drinking glass-shaped frustules; such pipes can form up to 35% of all forming frustules. Colchicine inhibits the formation of connecting spines, whereas paclitaxel causes spines to form a complex, branching shape. At the same time, inhibitors do not affect the formation of areolae (openings) in the frustule. We discuss the possibility that various processes of the diatom frustule morphogenesis are controlled by two different mechanisms: membrane-related micromorphogenesis and cytoskeleton-mediated macromorphogenesis.

## Abstract (precis)

**Summary:** It is shown that colchicine blocks mitosis and induces the formation of specific structures, lateral valves, in the diatom *Aulacoseira islandica*. Paclitaxel, on the other hand, doesn't have any effect on cell division.

## INTRODUCTION

Morphogenesis in different organisms is controlled by microtubules, actin microfilaments and their interaction, and is subject to similar rules. The shape of plant cells (Smith, 2003), trichome formation (Mathur, 2006), axon branching (Pacheco and Gallo, 2016), the multicellular process of neurotubule formation in vertebrates (Cearns et al., 2016) and the formation of haptophyte scales (Durak et al., 2017) are all controlled in similar ways. Microtubules are necessary for the orientation of cellulose fibers during cell wall synthesis in higher plants. They form the layer underlying the plasmalemma and take part in, among other processes, vesicular transport (Hardham et al., 1980; Lindeboom, 2012). Their inhibition can therefore cause various anomalies in the morphology of structures that are being formed, such as the *Arabidopsis thaliana* trichome (Mathur and Chua, 2000).

Many organisms utilize silica to build their cell walls and other structures at the nano- and micrometer scale (e.g. sponge spiculae, silicoflagellate skeletons, cysts and scales of chrysophyte algae; Müller, 2003). Diatoms, however, are most adept at silica-based morphogenesis; their cell walls are distinct in their morphological diversity and functionality (Mann et al., 2017).

Siliceous cell walls of diatoms are ornamented with micro- and nanometer perforations and processes, consisting of two valves (epitheca and hypotheca) and a system of overlapping girdle bands (Pickett-Heaps et al., 1990). Although the cell wall of diatoms is essentially an exoskeleton and serves to protect the cell from mechanical damage, including grazing (Hamm et al., 2003) and UV radiation (Aguirre et al., 2018), its elements form inside the cell and are excreted only after the morphogenesis is complete (Stoermer et al., 1965; Reimann et al., 1966; Crawford, 1981; Li and Volcani, 1984; Crawford and Schmid, 1986).

The valves in the cell wall are created after cytokinesis in a dedicated organelle called a silica deposition vesicle (SDV), formed by a specific membrane called the silicalemma. Girdle bands are created in a sequential manner during the interphase, each in a separate SDV (Drum and Pankratz, 1964; Reimann, 1964). For many centric diatoms, the valve synthesis has been shown to start at a small ring (annulus) in the middle of the valve face, from which radial siliceous ribs spread (Mann, 1984). During morphogenesis, the annulus is filled out by silica, the ribs elongate and branch, forming areolae and processes between them (Pickett-Heaps et al., 1990; Kalyuzhnaya, 2008; Tiffany and Hernández-Becerril, 2005; Sato, 2010).

Valve morphogenesis is known to be controlled in a significant part by the cytoskeleton, particularly by microtubules. In some species, microtubule inhibition has been shown to not completely stop valve morphogenesis, but rather to cause various structural anomalies, such as disordered areolae rows and curved raphe (Blank and Sullivan, 1983a,b; Cohn et al., 1989). In the centric diatom *Cyclotella cryptica*, exposure to 80 µg ml^−1^ colchicine causes the formation of ‘lateral valves’ (Oey and Schnepf, 1970), which are morphologically similar to normal valves, but shifted sideways from their normal position. The mechanism of their formation has not been explained. More recent studies have shown that microtubules determine the site where morphogenesis starts, the position of the forming valve relative to the mature one and the development of large frustule structures (Pickett-Heaps, 1998; Van de Meene and Pickett-Heaps, 2002, 2004; Tesson and Hildebrand, 2010a,b). In recent work, we have shown that exposing synchronized cultures of *Synedra acus* subsp. *radians* (Kützing) Skabitchevskii to colchicine and paclitaxel during certain morphogenesis stages produces specific deviations in the valve structure (Kharitonenko et al., 2015; Bedoshvili et al., 2017).

**Fig. 1.**
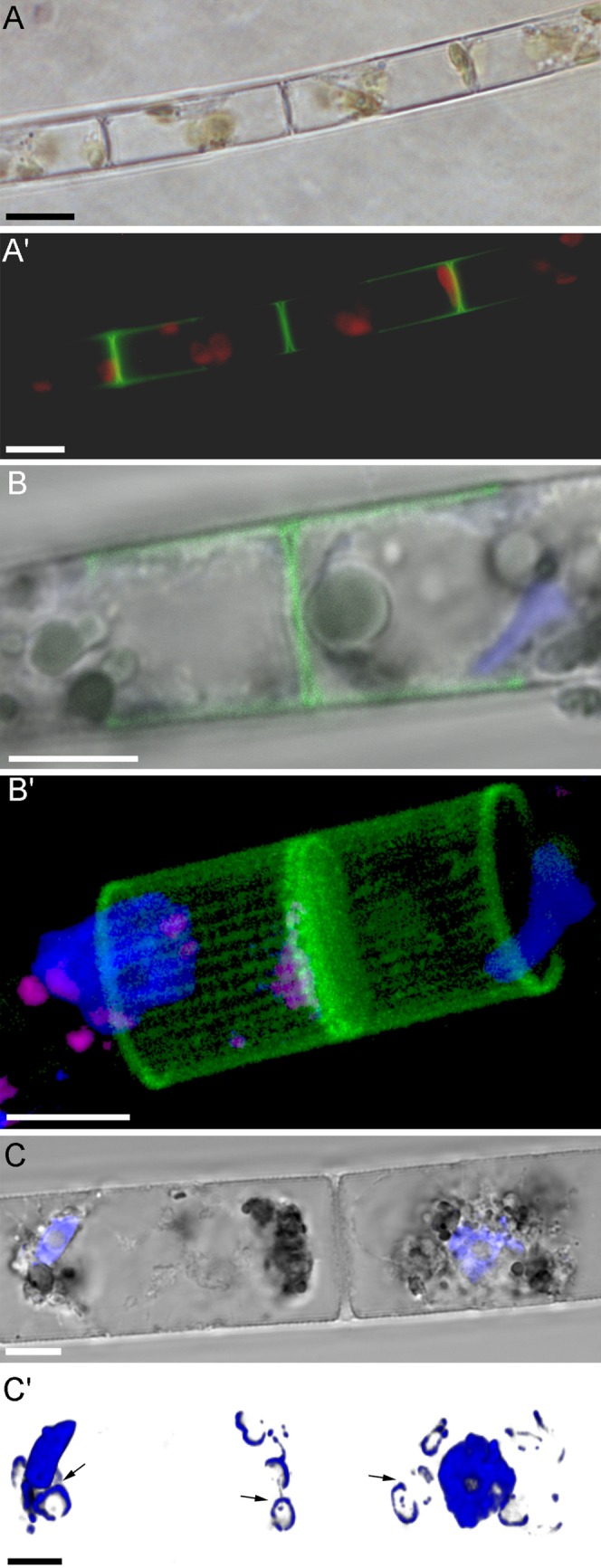
**Light (A), fluorescence (A′) and confocal (B-C′) images of *A.islandica* cells in culture.** (A,A′) Live cells in culture after Lysotracker Yellow HCK-123 staining. (B,B′) Cells fixed with paraformaldehyde (blue, nucleus; green, valves; purple, volutin). (C,C′) Fixed cells (blue, nucleus and DNA of chloroplasts). Scale bars: 10 µm (A,A′); 5 µm (B-C′).

**Fig. 2.**
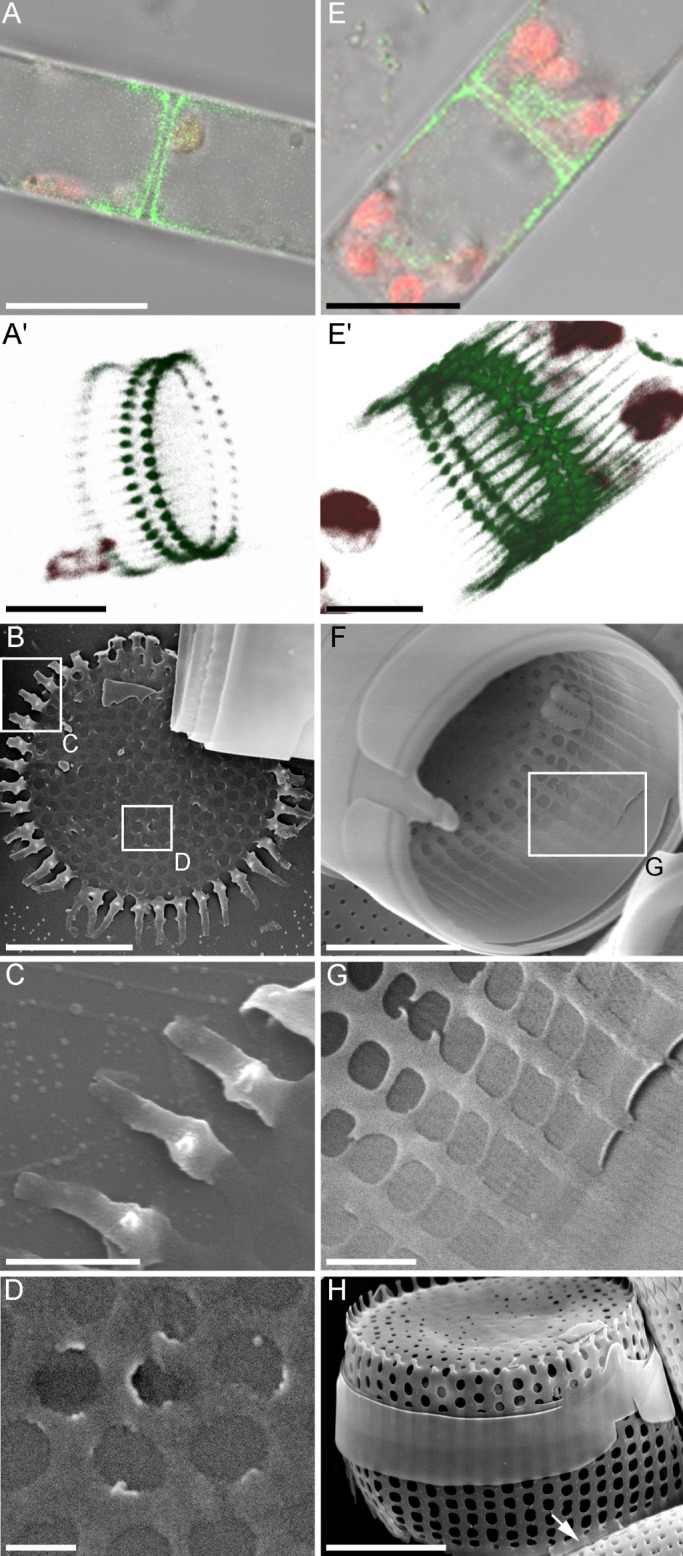
**Consecutive stages of valve morphogenesis in *A. islandica*.** (A-A′,E-E′) Laser scanning microscopy (LSM), staining by Lysotracker Yellow HCK-123 (green fluorescence) and autofluorescence of chloroplasts (red). (B-D,F-H) Scanning electron microscopy. (A-D) Valve plate with large openings at early stage of morphogenesis. (E-G) Valve with growing longitudinal bands. (H) Valve with formed mantle and collar (arrow). Scale bars: 10 µm (A,E); 5 µm (A′,E′,B,C,H); 1 µm (C,G); 500 nm (D).

**Fig. 3.**
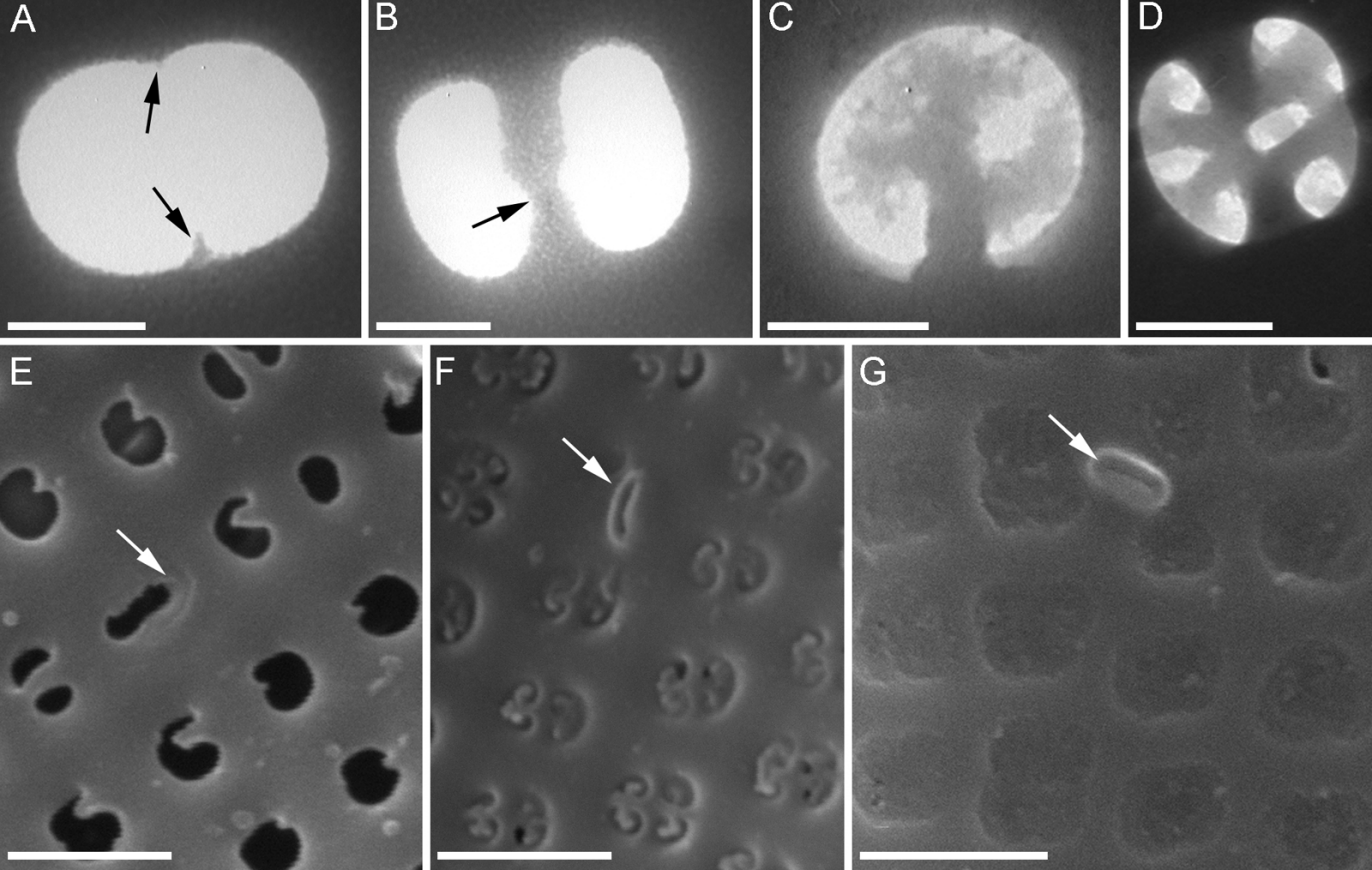
**Consecutive stages of areolae and rimoportulae formation in *A. islandica.*** [A-D, transmission electron microscopy (TEM); E-G, scanning electron microscopy (SEM)]. (A) Beginning of volae growth (arrows). (B) Volae connecting in the areolae middle (arrow). (C) Formation of areolae plate. (D) End stage of the velum formation. (E) Beginning of rimoportulae morphogenesis (arrow). (F) Thickening of rimoportulae (arrow). (G) Rimoportula on the mature valve (arrow). Scale bars: 200 nm (A,B,D); 100 nm (C); 1 µm (E-G).

The aim of the current work was to establish the morphogenetic stages of frustules in the diatom *Aulacoseira islandica* (Müller) Simonsen (a.k.a. *Aulacoseira skvortzowii* Edlund, Stoermer & Taylor), which possesses valves with a high mantle (Genkal and Popovskaya, 1991; Figs 1, 2 and 3), and to describe the anomalies in its frustule induced by microtubule inhibitors.

The Aulacoseiraceae family is one of the oldest freshwater diatom families (Ambwani et al., 2003). Drinking glass-shaped valves with high mantles are also common among ancient diatom fossils (Nikolaev and Harwood, 1999). In our opinion (Bedoshvili et al., 2012), the morphogenesis start site (annulus) in *Aulacoseira* spp. is not a small ring, but rather a border between the valve face and mantle, where the spines are located.

The genus *Aulacoseira* is present in many modern freshwater bodies all over the planet, including Lake Baikal, where it is often dominant in certain seasons (Popovskaya et al., 2011). Studying the morphogenesis of this genus is of considerable interest from an evolutionary point of view, as siliceous diatom valves can be preserved mostly intact in sediments for millions of years. Furthermore, the evolution of frustule morphology reflects the evolution of cellular morphogenetic mechanisms.

We used inhibitors that affect microtubules in different ways; colchicine blocks tubulin polymerization, whereas paclitaxel is an inhibitor of microtubule depolymerization. Based on the data acquired, we discuss various mechanisms by which silica deposition is regulated during morphogenesis of frustule elements.

## RESULTS

In culture, *A. islandica* cells formed filamentous colonies consisting of, on average, approximately six cells (although colonies of up to 17 cells were observed) that contain eight disc-shaped chloroplasts (Fig. 1A,A′). Dyeing with Lysotracker Yellow, aimed at visualizing forming valves, showed that in 2 days, each cell had divided and formed a regular, drinking glass-shaped valve (Fig. 2A′). Dyeing with DAPI showed that cells' nuclei exhibited a slightly concave, hemispherical shape (Fig. 1B,B′) and were orthogonal to neighboring cells. Chloroplast DNA was ring-shaped (Fig. 1C,C′) and DAPI dyeing also allowed us to detect polyphosphate volutin granules (Fig. 1B,B′).

### Morphogenesis of *A. islandica* valves

Morphogenesis of frustule elements was observed in *A. islandica* cells from natural samples taken during the spring bloom in 2006 and subsequently grown in a laboratory. We then identified the sequence of the morphogenetic states (Fig. 2). At the earliest detected stage, the forming valve is a plate with large, irregularly-placed openings of the future areolae on its surface and developing spines on the edges. The latter are, at this stage, the most silicified part of the valve (Fig. 2A-C). The longitudinal bands of the future mantle are forming near the spines (Fig. 2B,C).

During the following stages, these bands are growing and the areolae openings on the mantle are created by interlinking the bands (Fig. 2E-G). When the collar starts to form on the mantle's edge, the positions of future areolae on the mantle are already determined (Fig. 2H), and the fine structure of areolae and rimoportulae starts to develop (Fig. 3). The creation of velums that cover areolae openings starts from narrow volae that grow centripetally and connect in the middle of areolae (Fig. 3A,B). After they connect, the membrane, or areola plate, starts growing centrifugally (Fig. 3C,D). In mature valves, this membrane covers the entire areola opening (Fig. 3G). Numerous sedentary rimoportulae form from oval-shaped openings on the mantle (Fig. 3E-G); their thickening is simultaneous with velum volae formation and they assume their characteristic two-lipped shape at the same time as the velum membrane completes its development (Fig. 3G).

**Fig. 4.**
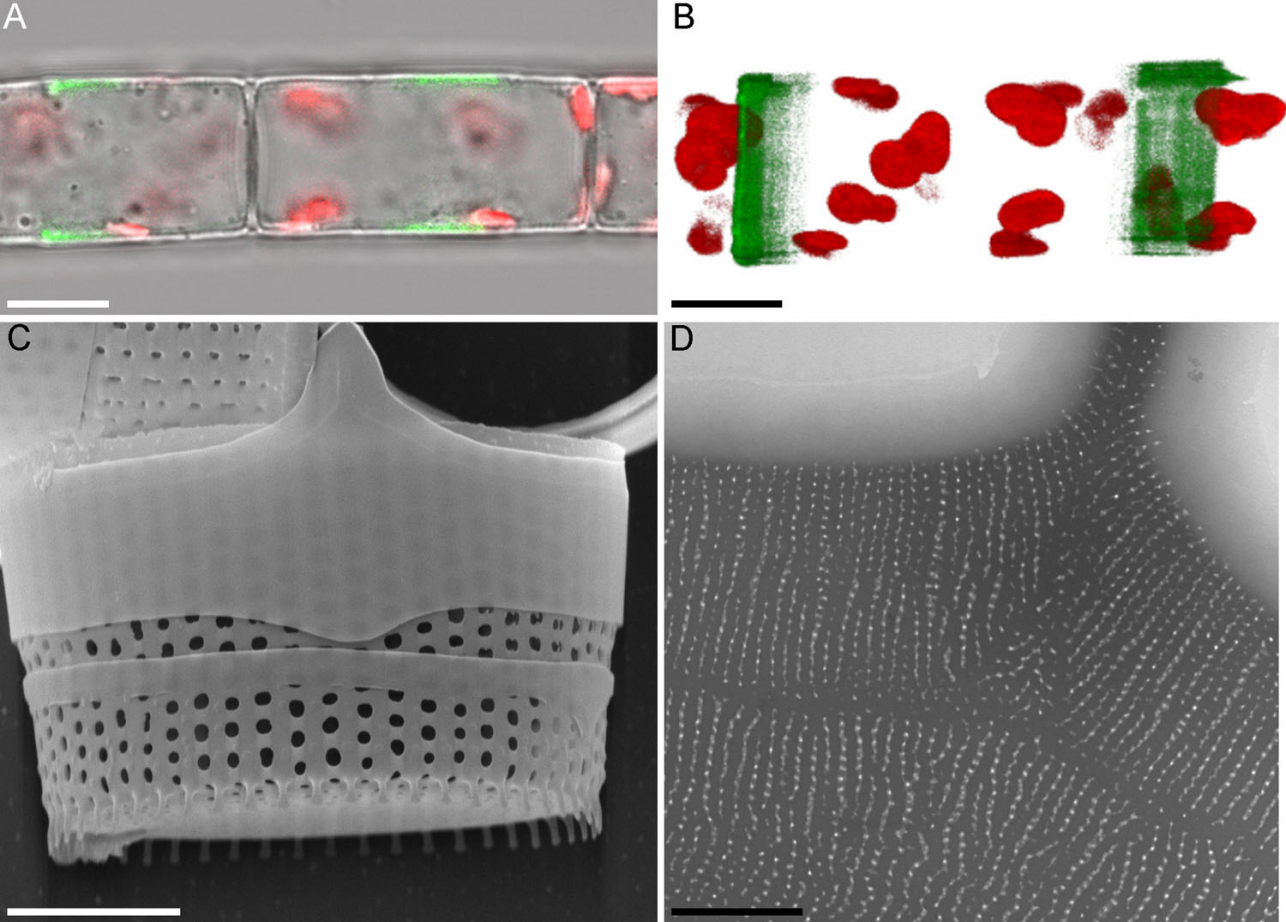
**The structure of the girdle bands in *A. islandica.*** [A-B, laser scanning microscopy (LSM); C, SEM; D, TEM]. (A,B) Girdle bands, visualized with Lysotracker Yellow HCK-123 (green fluorescence); red autofluorescence, chloroplasts. (C) Girdle bands on the immature valve. (D) Thin structure of the girdle band. Scale bars: 10 µm (A,B); 5 µm (C); 1 µm (D).

Fluorescent and confocal microscopy clearly visualized not only forming valves, but also thinner girdle bands (Fig. 4A,B), which are excreted from the cell and serve as a temporary mechanic protection during valve formation (Fig. 4C). Girdle bands exhibit ordered pore patterns that ensure their permeability, lightness and resilience (Fig. 4D).

### Colchicine and paclitaxel effects on valve morphogenesis in *A. islandica*

To estimate the effect of inhibitors on cultured cells, we used Lysotracker Yellow dye and counted the fluorescent valves after 3 and 5 days of cultivation. The proportion of such valves (relative to the total valve count) was a reliable method of studying both culture viability and major morphogenetic anomalies caused by varying inhibitor concentrations.

**Fig. 5.**
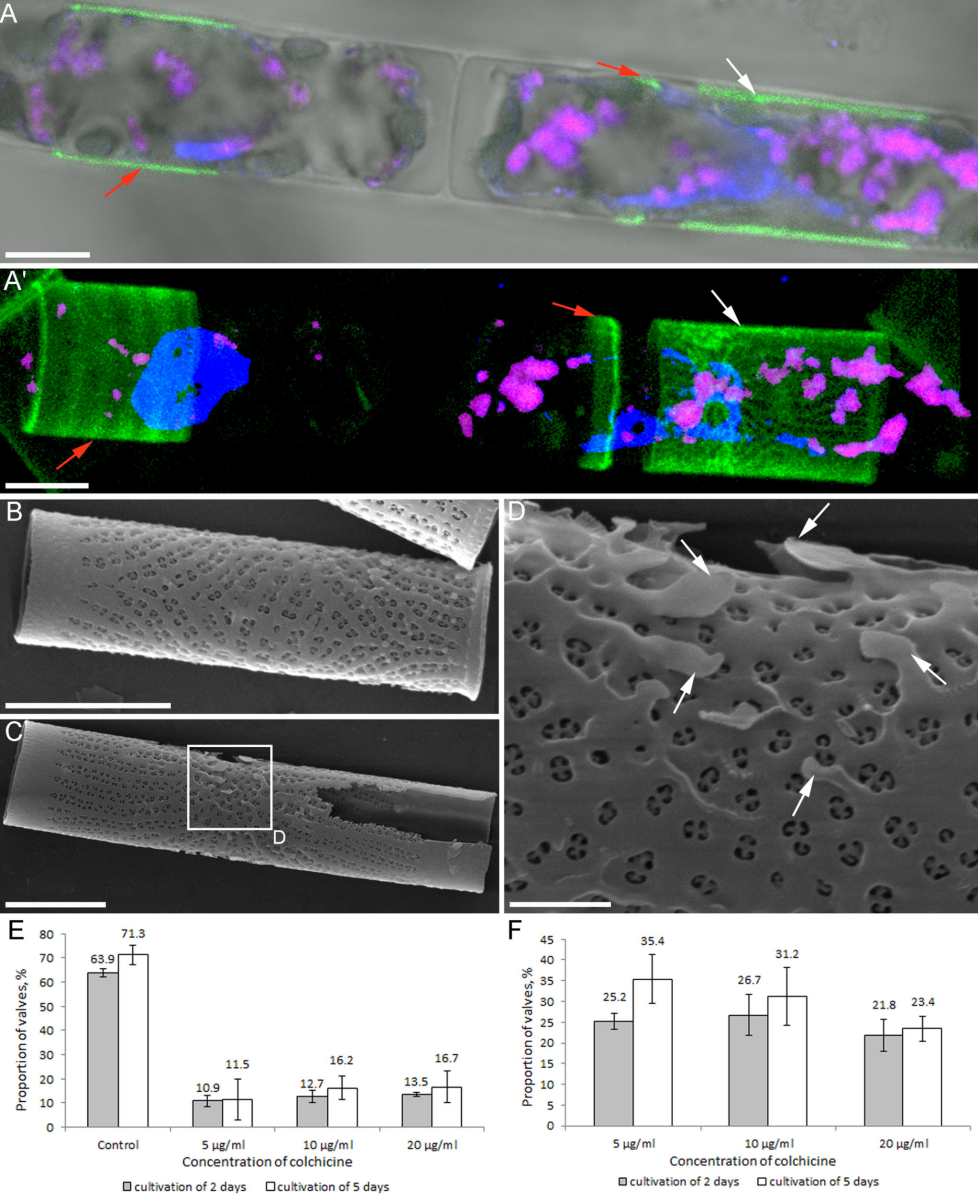
**The formation of lateral valves in *A. islandica* cells treated with colchicine.** (A,A′, LSM; B-D, SEM). (A,A′) Colony with lateral valve (white arrow) and girdle bands (red arrows). Blue fluoresces, DNA stained with DAPI; pink, volutin granules; green, Lysotracker Yellow HCK-123 in forming frustule elements. (B) Lateral valve with annulus down. (C) Lateral valve with annulus up. (D) An enlarged fragment of C. The deformed annulus is surrounded by spines (white arrows). (E) Change in the number of valves with the normal valve face. (F) The number of lateral valves in the experiment. Scale bars: 5 μm (A-C); 1 μm (D).

Confocal microscopy of the Lysotracker-dyed cells allowed us to detect lateral valves on the colonies (Fig. 5A,A′, right side). Cells with the lateral valves had a single, irregularly-shaped, elongated nucleus. In Fig. 5A and A′, a neighboring interphase cell can be observed actively forming girdle bands; its nucleus had two visible nucleoli (Fig. 5A,A′, left side). Scanning electron microscopy showed that a lateral valve has a single symmetry center, an annulus, which is surrounded by spines (Fig. 5B-D). Unlike a normal valve, the spines of a lateral valve cannot grow orthogonally to the valve face because of the space limitations imposed by girdle bands, resulting in them being pressed to the valve (Fig. 5D).

Under all studied colchicine concentrations and following cultivation for either 2 or 5 days, the proportion of normal (drinking glass-shaped) valves decreased approximately five-fold relative to the control (Fig. 5E). Lateral valves were found under all studied colchicine concentrations. Their maximal proportion was observed at a colchicine concentration of 5 µg ml^−1^ and a culturing time of 5 days; under these conditions, lateral valves comprised 35.4% of the total valve count (Fig. 5F).

**Fig. 6.**
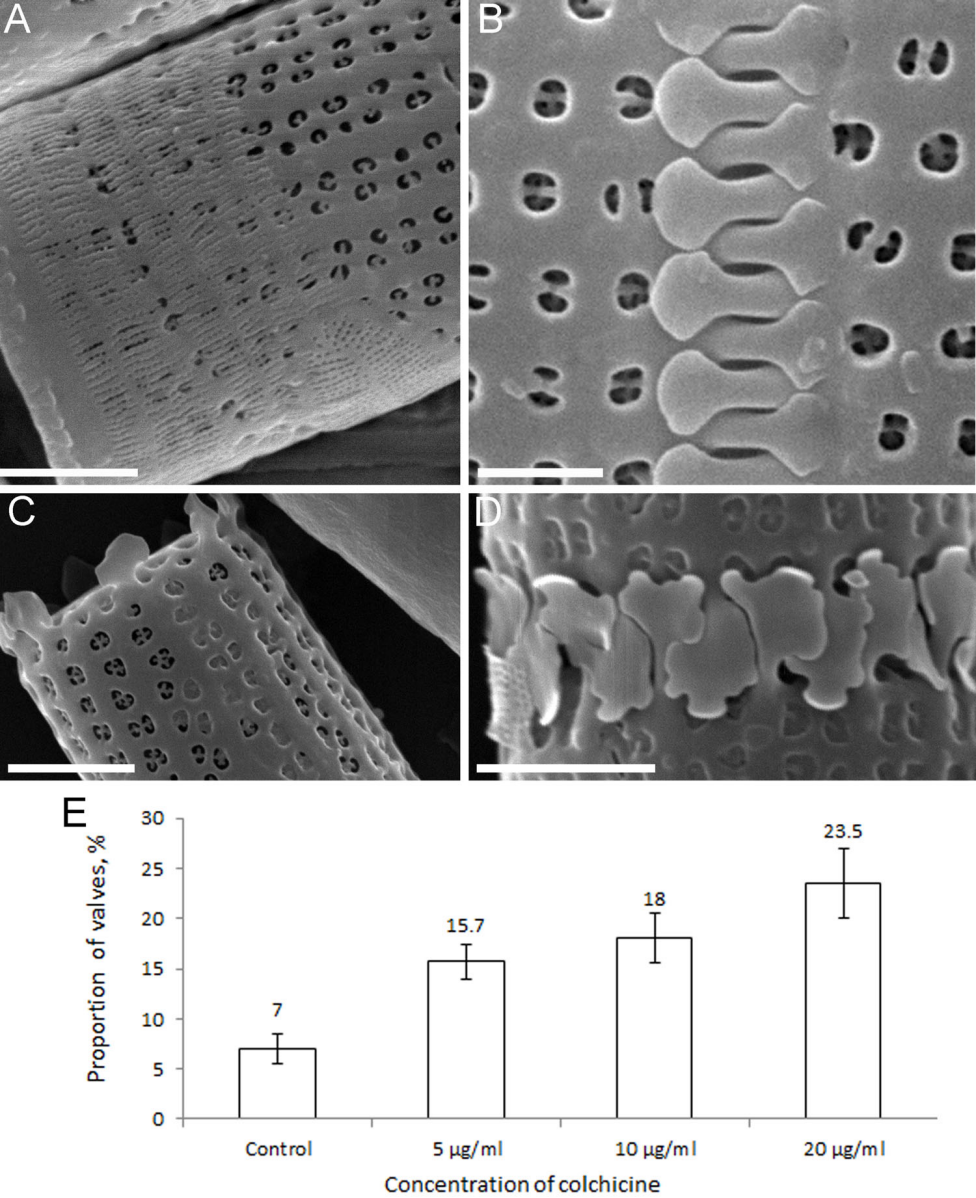
**Anomalies in the morphology of *A. islandica* spines after treatment of cells in culture with colchicine.** (A-D, SEM). (A) Several not completely silicified girdle bands on the valve surface. (В) Connecting spines in the control. (C) Change in the shape of the spines and their absence on the valve. (D) Change in the shape of spines. (E) Number of thin structure anomalies of spines according to SEM. Scale bars: 1 μm (A); 2 μm (B-D).

On the surface of 30±5.7% of frustules, we observed incompletely silicified girdle bands covering immature valves. Other anomalies in comparison to control cells (Fig. 6B) included deviations in spine shape or even complete absence of the spines (Fig. 6C,D). The proportion of valves with abnormal spines varied very little in relation to colchicine concentration, but was 15% higher than those of the control.

**Fig. 7.**
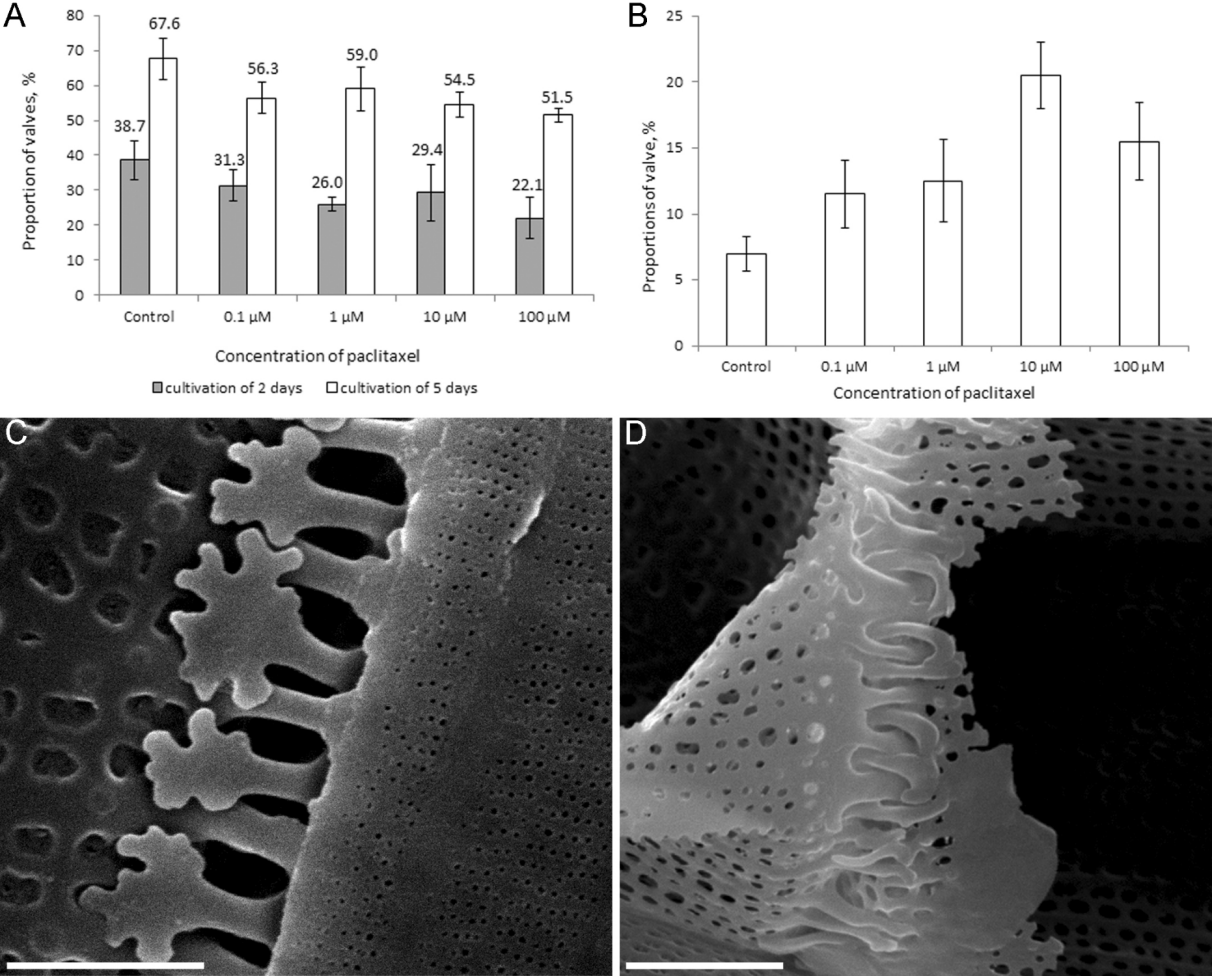
**Effects of paclitaxel on cells (C,D, SEM) in culture of *A. islandica*.** (A) The number of fluorescent valves in the control and experiment at 2 and 5 days after the start of the experiment. (B) The distribution of the number of spine anomalies, depending on the concentration of paclitaxel. (C,D) Spines with abnormal structure. Scale bars: 2 μm.

At the maximal paclitaxel concentration (100 μM), the proportion of forming valves was decreased by 20% relative to the control after 2 days of cultivation (Fig. 7A). By the fifth day, however, the proportion of forming valves nearly reached the control values. The number of ultrastructural anomalies increased with paclitaxel concentration (Fig. 7B), but these anomalies were similar at all concentrations; spines changed shape, becoming more branched (Fig. 7C) and, in some cases, narrow and curved (Fig. 7D).

## DISCUSSION

### Effects of inhibitors on cell division

To study the role of microtubules in valve morphogenesis, we selected two inhibitors with opposing effects. Colchicine is known to cause microtubule depolymerization, whereas paclitaxel stabilizes microtubules and prevents chromosome separation during cell division. Due to the latter effect, paclitaxel is an effective drug in oncotherapy (Rowinsky et al., 1993). In previous work, we observed the remarkable effect of both colchicine and paclitaxel on a pennate diatom, *Synedra acus* subsp. *radians* (Kharitonenko et al., 2015; Bedoshvili et al., 2017). Even at low concentrations, these reagents suppress division and cause various valve anomalies. In the current study, *A. islandica* cells proved more resistant to paclitaxel, as we did not find a concentration that would shut down division completely. Similarly, colchicine suppressed division in *A. islandica* at a concentration five times higher than that for *S. acus* subsp. *radians*.

Paclitaxel resistance has been observed in cancer cells as well. Despite decades of research, however, its mechanisms remain largely unknown. It has been shown that an important role in paclitaxel resistance is played by microtubule-associated proteins (MAPs) involved in mitotic chromosome separation (Shi and Sun, 2017). The microtubule organizing center of diatoms possesses features not observed in other organisms (Tippit and Pickett-Heaps, 1977; De Martino et al., 2009), and its molecular composition is nearly unstudied. Of the 11 MAPs that regulate paclitaxel activity, only two are found in complete diatom genomes (De Martino et al., 2009). The same results have been reproduced using currently-available diatom genomes and proteomes (∼70 species and strains in total). In this study, no homologs were found in diatoms for proteins of the Tau/MAP family, ninein-like protein or Transforming acidic coiled-coil-containing proteins (TACC). Curiously, a homolog of EB1 was found in the majority of diatoms, but it appears to have been absent from the genome of *S. acus* subsp. *radians*. Although no genome sequence is currently available for *A. islandica*, it is possible that *S. acus* is more sensitive to paclitaxel than is *A. islandica* (and, if this is true, most other diatoms) because of its absence.

### Spine formation under inhibitor treatment

The connection between microtubules and the formation of various valve processes during morphogenesis has been shown previously (Pickett-Heaps, 1998; Van de Meene and Pickett-Heaps, 2002; Tesson and Hildebrand, 2010a,b). For *A. islandica*, the most pronounced effects of both colchicine and paclitaxel were anomalies in spine structure (Figs 6 and 7). All species in the *Aulacoseira* genus form valves with spines that vary in shape and function. Cells are joined into colonies by connecting spines, whereas colonies split (and thus increase the population) using separating spines, usually conical in shape (Davey and Crawford, 1986; Bedoshvili et al., 2007). According to our data, spines are laid down at the earliest stages of valve formation, whereas their silification happens throughout most of the morphogenetic process and involves microtubules. At certain stages, connecting spines requires microtubular support, but if this support lasts for too long, spines start to branch, which they do not do in control or under colchicine treatment.

**Fig. 8.**
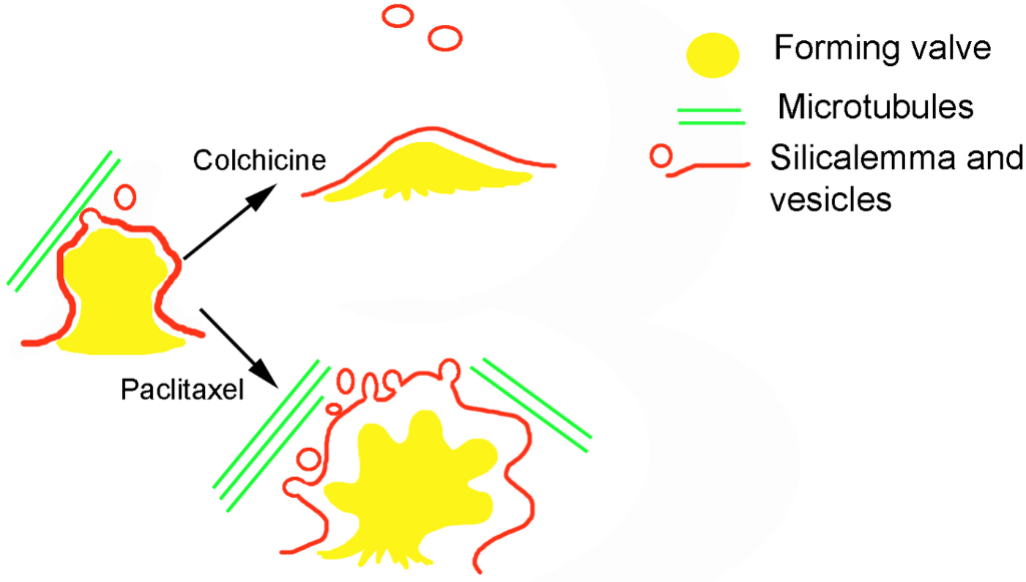
**Scheme of possible mechanism of microtubule inhibitor influence on the spine formation on the valve.**

The molecular biological mechanisms by which microtubules are involved in valve silification and formation of species-specific valve patterns are unknown. It is possible that morphogenesis is partially controlled by microtubule-mediated vesicular transport (Parkinson et al., 1999). The involvement of vesicular transport in valve morphogenesis is well supported (Dawson, 1973; Schmid and Schultz, 1979; Bedoshvili et al., 2018). It is possible that vesicles carry silification agents both as cargo (e.g. silaffins, silacidins or long-chain polyamines; Kröger et al., 1999; Kröger and Poulsen, 2008; Wenzl et al., 2008; Scheffel et al., 2011) and in their membranes (aquaporins, see Grachev et al., 2008; cingulins, see Tesson et al., 2017). During the paclitaxel treatment, the concentration of these agents increased, causing deviations in spine shape (Fig. 8).

Interaction of the two sister cells during the morphogenesis of their valves has been discussed previously (Mann, 1984), but to date, this aspect of morphogenesis remains understudied. It would be reasonable to assume that two sister *A. islandica* cells closely interact during connecting spine development, because later these spines keep the cells together. Nevertheless, spines being positioned on the lateral valves means that the sister cell is not necessary for their development.

### Lateral valve formation

It has been suspected that microtubules determine SDV localization at certain stages of morphogenesis (Tesson and Hildebrand, 2010a). However, even after colchicine-induced microtubule depolymerization, SDV remained on the periphery of the cell, apparently in close contact with the plasmalemma. Thus, the position of SDV is most likely controlled not only by microtubules, but also by mechanisms that are unknown so far with the participation of transmembrane proteins (e.g. cingulins – Tesson et al., 2017), which keep the plasmalemma and silicalemma side by side. If this were not the case, we would likely observe at least some abnormal valves forming in the middle of the cell. Our data show that even if karyokinesis does not happen, a single lateral valve forms near the (presumed) cytokinesis site, directly under the plasmalemma. Due to the existing cell program, the morphology of the lateral valve has features of the normal valve with rows of areolae, spines and perforated plate of annulus, but they are deformed due to the abnormal location of the annulus.

It is obvious that in our study, the proportion of lateral valve-carrying cells in the culture reflected the proportion of cells undergoing mitosis at the moment of colchicine treatment. This contradicts the results of an earlier work (Oey and Schnepf, 1970), wherein light microphotographs of *C.* *cryptica* cells with dyed nuclei and, presumably, lateral valves clearly showed the presence of the two nuclei. It is probable that *A. islandica* and *C. cryptica* are remarkably different regarding their mechanisms of chromosome separation and determination of the valve symmetry center.

### Membrane-mediated morphogenetic processes in valve morphogenesis

Presence and correct positioning of microtubules is necessary for areolae formation (Blank and Sullivan, 1983a; Cohn et al., 1989; Kharitonenko et al., 2015; Bedoshvili et al., 2017). Past microtubule inhibition experiments have shown that colchicine exposure at certain stages of morphogenesis in *S. acus* subsp. *radians* causes areolae to be occluded with silica (Kharitonenko et al., 2015), whereas paclitaxel addition produces abnormal areola and velum structures (Bedoshvili et al., 2017). We expected these inhibitors to have a similar effect on *A*. *islandica* cells, but this was not the case. Electron microscopy showed no effect of microtubule inhibitors on either areolae or velum structure, with the exception of disordered areolae rows, which are mediated by atypical annulus position in lateral valves. Valves with disordered areolae rows are occasionally created naturally, and we were unable to increase their proportion by exposing cells to microtubule inhibitors. This phenomenon can be caused by the existence of two distinct morphogenetic control mechanisms proposed earlier (Pickett-Heaps et al., 1990; Wetherbee et al., 2000; Hildebrand and Wetherbee, 2003). Control of valve morphogenesis was divided in those previous works into membrane-related morphogenesis (micromorphogenesis) and macromorphogenesis. The latter term was proposed due to the effect of the organelles or cytoskeleton structures responsible for the shape and position of the SDV and some large-scale valve elements. The observed effect of raphe presence and location on the position of the microtubule organizing center in some pennate diatoms (Pickett-Heaps et al., 1988, 1990; Schmid et al., 1996) could be an example of macromorphogenetic control, as could be the development of the labiate process, which in some centric diatoms is controlled by the labiate process apparatus, closely ontogenetically related to the spindle apparatus and post-mitotic microtubule system (Pickett-Heaps et al., 1988). Valve micromorphogenesis, on the other hand, means that the intra-SDV environment, silicalemma and, to a lesser degree, neighboring plasmalemma affect the silica deposition processes. This, hypothetically, is how the creation of pore fields and areolae velums is regulated.

It is necessary to separate these mechanisms in order to understand the morphogenesis of diatom valves. The lack of a microtubule inhibitor effect on the position and structure of areolae on forming *A. islandica* valves shows that their development, at least in this species, is under micromorphogenetic control, with microtubules playing a minor role. It is known that silica deposition during the formation of similar velums can occur spontaneously (Gordon and Drum, 1994; Kröger and Poulsen, 2008), whereas girdle band synthesis relies on microtubule-dependent transport, as evidenced by the decreased silification and morphological deviations in the girdle bands under colchicine treatment (Fig. 6B).

Diatoms' ability to create a wide diversity of siliceous structures could be a product of two interacting control systems, both cellular and biochemical silification control. This could be one of the factors that has allowed this group to evolve and spread to a very broad range of environments.

## MATERIALS AND METHODS

### Sampling and cell cultures

The *A. islandica* culture was isolated from a natural population in Lake Baikal and cultivated in the DM medium (Thompson et al., 1988) at 4°С with natural light and day–night cycle.

### Treatment of the culture with colchicine and paclitaxel

Assessment of the impact of colchicine and paclitaxel on cells of *A. islandica* was performed via progressive addition of colchicine (Sigma-Aldrich) or paclitaxel (Sigma-Aldrich) to the medium to a final concentration of 5, 10 or 20 µg ml^−1^ for colchicine, and 0.1, 1, 10 or 100 µM for paclitaxel. Lysotracker Yellow H-123 viable dye (Thermo Fisher Scientific) was added simultaneously to a final concentration of 0.3 µМ (Declés et al., 2008). All experiments were conducted in triplicate.

### Fluorescence microscopy and scanning laser microscopy

The cells were studied by means of fluorescence microscopy and scanning laser microscopy after 2 and 5 days of exposure to inhibitors and Lysotracker Yellow H-123. The number of fluorescent cells was counted within 100 cells encountered. The cells were studied with the Axiovert 200 incident light microscope (Zeiss, Oberkochen, Germany) equipped with a blue filter for light 546 nm in length. Imaging was conducted by means of the Pixera Penguin 600CL (Pixera, Bourne End, UK) video camera and the VideoTest 5.0 software package (Akond, https://www.akondphoto.ru/?issue_id=75).

For the scanning laser microscopy, cells of *A. islandica* were cultivated in the presence of colchicine (20 μg ml^−1^) and Lysotracker Yellow H-123 fluorescent dye over 2 days, fixed with paraformaldehyde (4%) in 0.066 М phosphate buffer (рН 7.4) for 30 min and then washed with the same buffer twice. This was followed by DAPI (10 μg ml^−1^; Sigma-Aldrich) staining for 10 min. After this procedure, the cells were rinsed twice with the buffer and placed in Prolong Gold antifade (Thermo Fisher Scientific). A LSM 710 confocal microscope (Zeiss) equipped with Plan-Apochromat 63×/1.40 Oil DIC M27 immersion lens (Zeiss) was used for the cell study. Lysotracker Yellow H-123 fluorescence was excited with a 488 nm laser; its emission was registered in the range of 496–647 nm. DAPI fluorescence was excited with a 405 nm laser; its emission was registered in the ranges of 410-492 nm (DNA dyeing) and 569-603 nm (volutin dyeing). The images obtained were processed with ZEN 2010 software (Zeiss). 3D-reconstructions for Fig. 1B and C were obtained from 100 optical sections (thickness on the z axis: 12.5 μm); for Fig. 2A′ and E′ from 534 optical sections (thickness on the z axis: 15 μm); for Fig. 5A′ from 150 optical sections (thickness on the z axis: 15 μm). The images were minimally processed in the ZEN 2010 program: shown in transparent mode with the maximum channel threshold for noise elimination of 10 units.

### Frustule purification for electron microscopy

Electron microscopy requires cleaning the frustules of organic material. The cells were boiled in three changes of 6% SDS solution (0.5 h each), then washed five times with deionized water and centrifuged. Concentrated nitric acid (Reactiv, Novosibirsk, Russia) was added to the pellet and the mixture was incubated in a water bath (95°C) for 1 h, washed in three portions of ethyl alcohol, treated with concentrated hydrochloric acid for 24 h and then washed in at least five portions of distilled water. After each step, the material was pelleted by centrifugation at 1000×***g*** for 10 min.

### Transmission electron microscopy

Frustule suspensions were placed on copper grids with formvar support and examined using a Leo 906 Е electron microscope (Zeiss) at an acceleration voltage of 80 kV. Micrographs were made with the Mega View II Zeiss camera.

### Scanning electron microscopy (SEM)

Suspensions of cleaned frustules were pipetted onto cover glasses, dried and mounted on SEM stubs with carbon double-sided adhesive tape (SPI Supplies, West Chester, USA). Morphological analysis among 200 frustules encountered was carried out using a QUANTA 200 scanning electron microscope (FEI Company, Hillsboro, USA).

### Search for the homologs of paclitaxel-related proteins

Complete sets of proteins predicted based on diatom genomes and proteomes were searched for the homologs of target proteins via hmmer 3.1b2 (Eddy, 2011) using PFAM hidden Markov models (Finn et al., 2014).
